# Protective Effects of Ganoderma Triterpenoids Supplementation Against Myocardial Injury in Exhaustion Exercise Mice via Regulation of the Keap1/Nrf2/HO‐1 Pathway

**DOI:** 10.1002/fsn3.70937

**Published:** 2025-09-12

**Authors:** Jingrong Li, Ling Zhang, Guangfeng Qin, Weiguo Liu, Xin Xu, Jialin Zhu, Shengmei Zhao, Taotao Qiu

**Affiliations:** ^1^ Physical Education and Health Guangxi Normal University Guilin China; ^2^ College of Sports and Health Guangxi College of Sports Education Nanning China; ^3^ School of Physical Education South China Normal University Guangzhou China

**Keywords:** anti‐fatigue, *ganoderma lucidum* triterpenoids, myocardial injury, oxidative stress

## Abstract

Exhaustive physical exercise, while promoting cardiovascular fitness, can paradoxically lead to excessive oxidative stress, systemic fatigue, and myocardial injury. Despite increasing awareness of exhaustion exercise myocardial damage, effective preventive strategies remain limited. Natural bioactive compounds with antioxidant and antiapoptotic properties have gained attention as potential interventions. Among them, triterpenoids derived from *Ganoderma lucidum* triterpenoids (GLTs) are notable for their potent free radical scavenging and cytoprotective effects. However, their cardioprotective potential under conditions of exhaustion exercise oxidative stress has not been fully elucidated. This study explores the antifatigue potential of GLTs and elucidates their underlying mechanisms through the establishment of a mouse model of exhaustion exercise via exhaustive treadmill running. This study showed that GLTs significantly alleviated exhaustion exercise by reducing serum fatigue biomarkers (CK, BUN, and LDH) and improving myocardial histopathological conditions. GLTs enhanced antioxidant capacity by decreasing MDA levels, increasing SOD and CAT activities, and elevating GSH content, thereby mitigating oxidative stress. Additionally, GLTs regulated apoptosis by downregulating Bax and Caspase‐3 expression, upregulating Bcl‐2 levels, and reducing the Bax/Bcl‐2 ratio. Mechanistically, these effects were associated with activation of the Keap1/Nrf2/HO‐1 signaling pathway. Collectively, this study provides new insights into the context‐specific role of GLTs in protecting against exhaustion exercise myocardial injury. Our findings highlight the therapeutic potential of GLTs as a natural antioxidant strategy for mitigating oxidative stress, delaying fatigue, and preserving cardiac function under conditions of intensive physical exertion.

## Graphical abstract

Ganoderma lucidum triterpenoids (GLTs) demonstrate significant antifatigue and cardioprotective effects in a mouse model of myocardial injury induced by exhaustive exercise. Administration of GLTs attenuated oxidative stress, inhibited apoptosis, and maintained myocardial structural integrity, primarily through activation of the Keap1/Nrf2/HO‐1 signaling pathway and enhancement of endogenous antioxidant defenses. These findings identify GLTs as a promising, dose‐dependent natural intervention for mitigating myocardial damage associated with exhaustive exercise.

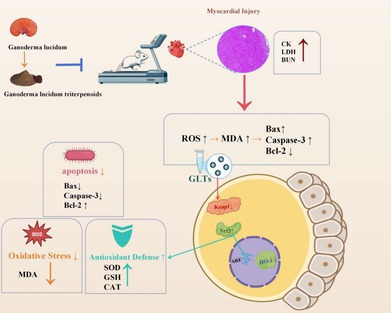

## 1 Introduction

Physical exercise offers significant health benefits by improving cardiovascular function, strengthening the immune system, and lowering the risk of chronic diseases (Axtamov 2024). However, exhaustion exercise fatigue is characterized by a decline in the body's ability to maintain stable physiological function or exercise intensity, primarily affecting motor tissues and organs. When the intensity or duration of exercise surpasses physiological limits, it may trigger a range of adverse responses (Bestwick‐Stevenson et al. 2022; Ma et al. 2020; Manjaly et al. 2019; Matei et al. 2022). The heart is particularly vulnerable, with excessive exertion potentially altering cardiac morphology, impairing function, and increasing the risk of arrhythmias, heart failure, and exhaustion exercise myocardial injury (Leischik et al. 2020; Stewart et al. 2016). In contrast to pathological cardiac injury, exhaustion exercise myocardial damage involves reversible oxidative and apoptotic stress without overt necrosis; yet, it remains a largely underexplored area despite its growing relevance. Addressing such damage requires targeted interventions. Myocardial injury involves oxidative stress (Nie et al. 2010), calcium dysregulation (Joviano‐Santos et al. 2019), inflammation (Chauin 2021), apoptosis (D'Oria et al. 2020), and endoplasmic reticulum stress (Han et al. 2023), collectively contributing to cardiomyocyte dysfunction and cardiovascular disease. Among these, oxidative stress and apoptosis are key drivers (Wang et al. 2018; Zhang, Luo, et al. 2022), as exhaustive exercise increases free radicals, triggering apoptosis via specific pathways (Carvalho et al. 2020; Wu, Liu, et al. 2022). Nuclear Factor Erythroid 2‐Related Factor 2 (Nrf2), a crucial transcription factor in antioxidant defense, helps to maintain redox homeostasis and counteract oxidative damage (Baird and Yamamoto 2020; Lin et al. 2018; Liu et al. 2022). Its activation has been shown to protect cardiomyocytes from oxidative stress‐induced injury, underscoring its potential therapeutic significance (Shen et al. 2018).

In recent years, there has been growing interest in natural plant‐derived compounds as potential interventions to combat exhaustion during exercise, primarily through enhancing antioxidant defenses and modulating immune function (Hu et al. 2020; Tuo et al. 2021; Zhang 2020). Ganoderma lucidum, a traditional Chinese medicinal herb, contains Ganoderma triterpenoids (GLTs) as its primary active constituents, which exhibit potent antioxidant, anti‐inflammatory, and immunomodulatory properties (Feng et al. 2013; Li et al. 2022; Zhang et al. 2023). Prior studies demonstrated GLTs can mitigate oxidative stress and inflammation via pathways such as NF‐κB and MAPK, contributing to protective effects in various tissues (Hsu et al. 2018; Xu et al. 2021; Zhang, Lv, et al. 2022). Furthermore, GLTs have been shown to promote fatty acid oxidation and exert antifatigue effects during physical exertion (Tang 2020).

However, despite these promising findings, the specific cardioprotective effects of GLTs in the context of exhaustive exercise myocardial injury remain inadequately elucidated. This type of injury, primarily driven by transient oxidative stress and apoptosis without overt necrosis, differs fundamentally from acute pathological models such as myocardial infarction or drug‐induced cardiotoxicity. Instead, it reflects the cumulative myocardial stress experienced by athletes or individuals subjected to prolonged or repeated physical exertion. These physiological distinctions highlight both the clinical significance and the innovative value of utilizing an exhaustive exercise model to investigate the cardioprotective potential of GLTs.

Therefore, we hypothesized that GLTs could mitigate exercise‐induced myocardial injury in fatigued mice by modulating the Keap1/Nrf2/HO‐1 signaling pathway. To test this, we established a robust treadmill‐based exhaustive exercise model and investigated the protective effects and underlying mechanisms of GLT intervention.

## 2 Materials and Methods

### 2.1 Materials and Chemicals

The GLTs used in this experiment were extracted in the laboratory, with a measured purity of 73.17%. ELISA kits for Creatine kinase (CK), Lactate dehydrogenase (LDH), Blood urea nitrogen (BUN), Superoxide dismutase (SOD), Malondialdehyde (MDA), and Glutathione (GSH) were provided by Quanzhou Leida Qibo Biotechnology Co. Ltd., and the Catalase (CAT) detection kit was supplied by Nanjing Jiancheng Bioengineering Institute. The mammalian protein extraction kit, nuclear‐cytoplasmic protein preparation kit, protease inhibitor, phosphatase inhibitor, PMSF, and loading buffer were purchased from Shanghai Beyotime Biotechnology Co. Ltd. Tween‐20, Tween‐80, glycine, SDS, Tris‐base, and acrylamide were obtained from Beijing Solebo Technology Co. Ltd., while protein standards were sourced from Hangzhou Ford Biotechnology Co. Ltd. HRP‐labeled goat antirabbit and goat antimouse secondary antibodies, along with Kelch‐like ECH‐associated protein 1 (Keap1), Nuclear factor erythroid 2‐related factor 2 (Nrf2), BCL2‐associated X Protein (Bax), Lamin B1 Monoclonal Antibody (LaminB1), and Glyceraldehyde‐3‐phosphate dehydrogenase (GAPDH)antibodies, were provided by Wuhan Sanying Biotechnology Co. Ltd. The SOD‐1 antibody was sourced from Wuhan Fine Biotech Co. Ltd., and the B‐cell lymphoma‐2 (Bcl‐2) antibody was obtained from Affinity Biosciences, USA. The Heme oxygenase‐1 (HO‐1), Catalase, and Cysteine aspartate protease‐3 (Caspase‐3) antibodies were obtained from Beyotime Biotechnology, Shanghai, China. Prestained protein markers, BCA protein concentration kits, and SDS‐PAGE gel kits were purchased from Shanghai Yeasen Biotechnology Co. Ltd. TBS crystals were supplied by Fuzhou Feijing Biotechnology Co. Ltd., and the ECL chemiluminescence solution was provided by Beijing LandJerk Technology Co. Ltd.

### 2.2 Animal Grouping and Handling

Sixty KM male mice were purchased from Hunan Slyke Jingda Laboratory Animal Co. (License No: SCXK (Xiang) 2019‐0004). They were kept in the laboratory animal room of the College of Physical Education and Health of Guangxi Normal University and passed the review of the Ethics Committee of Guangxi Normal University (Acceptance code of ethics review: GXNU‐202306‐001). The indoor temperature was kept at 25°C ± 1°C, and 12 h of light, SPF grade feed, and pure water were provided every day. After 1 week of acclimatization, the mice were randomly divided into five groups (*n* = 12 per group): control group (no treatment), exhaustive exercise group (model), and three GLTs intervention groups receiving low (100 mg/kg), medium (200 mg/kg), and high (300 mg/kg) doses, respectively.

The dosage of GLTs for intragastric administration was established based on previous studies (Shi et al. 2022; Wang et al. 2021; Yuan 2020), as well as the recommended usage of Ganoderma lucidum in the Chinese Pharmacopeia (Chinese Pharmacopoeia Commission 2020). The final dosing regimen (100, 200, and 300 mg/kg) was adjusted using standard interspecies dose conversion principles for laboratory animals (Li and Zhang 2023). These dose levels were selected after pilot testing and were well‐tolerated by mice throughout the experiment, with no signs of toxicity or adverse effects observed. Mice in the model and GLTs groups received daily GLTs gavage 90 min prior to treadmill training, while the control group received no intervention. The model group was simultaneously administered intragastric saline.

### 2.3 Establishment of Exhaustion Exercise Myocardial Injury Model and Training Protocol

The treadmill protocol was adapted from Zhang (Zhang et al. 2021) to simulate myocardial stress caused by sustained high‐intensity exercise. Mice in the GLTs groups received GLTs by gavage 90 min before training, followed by 60 min of rest. The model group was simultaneously administered physiological saline via gavage, while the control group received no treatment. All groups underwent a 7‐day adaptation (10 m/min, 30 min/day). Formal training included running on a 10°incline at 10 m/min for 10 min, 15 m/min for 10 min, and then 20 m/min until exhaustion. This protocol is widely used to induce systemic fatigue and early myocardial changes, such as oxidative stress and apoptosis, without causing overt necrosis. Mice trained 6 days/week for 6 weeks to simulate cumulative physiological cardiac load.

Exhaustion was defined as the inability to maintain pace, marked by the mouse remaining in the rear one‐third of the treadmill for three consecutive instances despite visual, auditory, or tactile stimulation (Arullampalam et al. 2023).

### 2.4 Electrocardiogram Examination

Electrocardiogram (ECG) examination was performed 24 h after the end of 7 weeks of treadmill exercise. After anesthesia with isoflurane, electrocardiogram detection was performed in mice without activity or anesthesia response. The Powerlab physiological recorder was used to detect the mouse ECG, and the acquired ECG was analyzed using LabChart 7.2 software (Zhang, Luo, et al. 2022).

### 2.5 Sampling and Sample Preparation

Each mouse was intraperitoneally injected with 1.25% tribromoethanol solution at 0.1 mL/10 g. After blood sampling from the eyeballs, the blood was left to stand for 24 h to separate the serum, which was used for biochemical indicators. The abdominal cavity was exposed, then the thoracic cavity was opened, then the heart was isolated, and the mouse heart was rinsed in precooled normal saline. One‐third of the heart near the apex was used for protein detection experiments and placed in a refrigerator at −80°C for subsequent detection, while the rest was fixed with 10% neutral formaldehyde for pathological experiments.

### 2.6 Detection of Blood Biochemical Indicators

The blood was centrifuged at 4000 r/min for 20 min, and the supernatant serum was taken. Mouse serum creatine kinase (CK), lactic dehydrogenase (LDH), and blood urea nitrogen (BUN) levels were detected by the kit method. For mouse serum, catalase (CAT) activity was measured by the visible light method, superoxide dismutase (SOD) activity was detected using the WST‐8 colorimetric assay, and malondialdehyde (MDA) activity was determined by the thiobarbituric acid colorimetric method. The determination of reduced glutathione (GSH) was made by measuring the absorbance of reduced glutathione GSH reacting with DTNB to form complexes.

### 2.7 Histopathological Analysis

Histological procedures followed Wu's protocol (Wu et al. 2021). Hearts were fixed in 10% neutral formaldehyde, dehydrated, paraffin‐embedded, and sectioned at 4 μm. After H&E staining, myocardial morphology was assessed under light microscopy. To enhance evaluation objectivity, a semiquantitative scoring system was used, assessing (1) myofiber disorganization, (2) necrosis or vacuolization, and (3) inflammatory infiltration, each rated 0–4 (0 = none, 4 = extensive). Total scores ranged from 0–12. Two blinded investigators independently evaluated all sections, and the mean score was used for analysis. This scoring system quantified myocardial injury severity and evaluated the protective effects of GLTs.

### 2.8 Western Blot

The method followed Chen (Chen et al. 2022), total protein was extracted from frozen heart tissue using cold lysis buffer with protease inhibitors. Cytoplasmic and nuclear proteins were isolated using a protein extraction kit, and protein concentrations were determined by the BCA assay. Equal protein amounts (20‐40 μg) were separated by 10% SDS‐PAGE and transferred to PVDF membranes. After blocking with 5% milk in TBST, membranes were incubated with primary antibodies overnight at 4°C. HRP‐conjugated secondary antibodies were applied for 45 min at 37°C. Protein bands were detected using ECL and analyzed with ImageJ. Expression levels were normalized to β‐actin, GAPDH, or LaminB1.

### 2.9 Statistical Analyses

The experimental data in this study were statistically analyzed by SPSS 26.0 software, expressed by mean ± standard deviation, and statistically analyzed by one‐way analysis of variance (ANOVA). When *p* < 0.05, the difference was statistically significant. The statistical charts were obtained by Graphpad Prism 7.0.0 software. ECG was analyzed using LabChart 7.2 software, and Western blot results were obtained using the Image J software.

## 3 Results

### 3.1 Effect of GLTs on Fatigue Index in Exhaustion Exercise Mice

**FIGURE 1:**
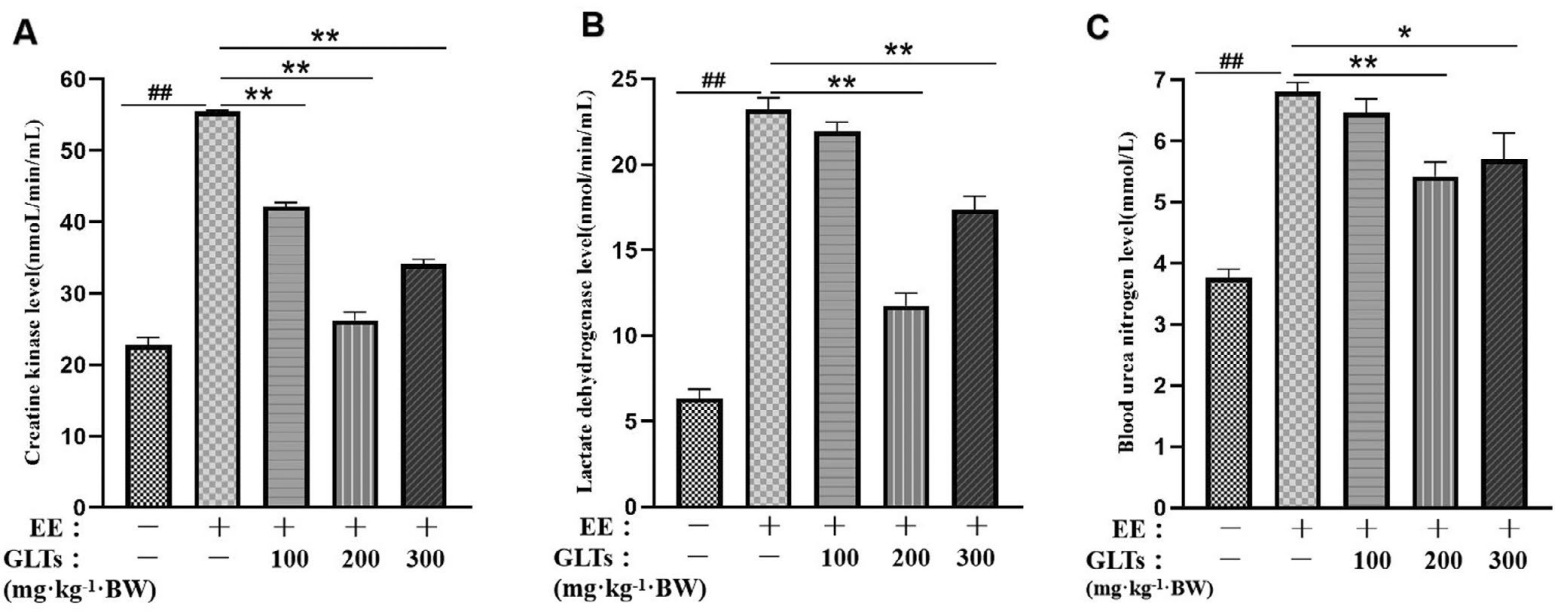
Effect of GLTs on exhaustive exercise‐induced fatigue in mice. (A) Creatine kinase (CK) level. (B) lactate dehydrogenase (LDH) level. (C) Malondialdehyde (BUN) level. ##*p* < 0.01 compared with the control group. *p < 0.05 compared with the model group. **p < 0.01 compared with the model group. (x ± SD, *n* = 12).

Exhaustive exercise resulted in a significant increase in fatigue‐associated biomarkers, including CK, LDH, and BUN, confirming the successful establishment of the fatigue model. As shown in Figure 1, the levels of these biomarkers were markedly elevated in both the model and GLT‐treated groups compared with the control group. However, GLT supplementation, particularly at the moderate dose, significantly reduced CK, LDH, and BUN levels. The GLTs‐M group exhibited values most comparable to those of the control group. These results suggest that GLTs may alleviate exhaustion from exercise by attenuating related biochemical changes.

### 3.2 Effect of GLTs on Running Exhaustion Time of Mice

**FIGURE 2:**
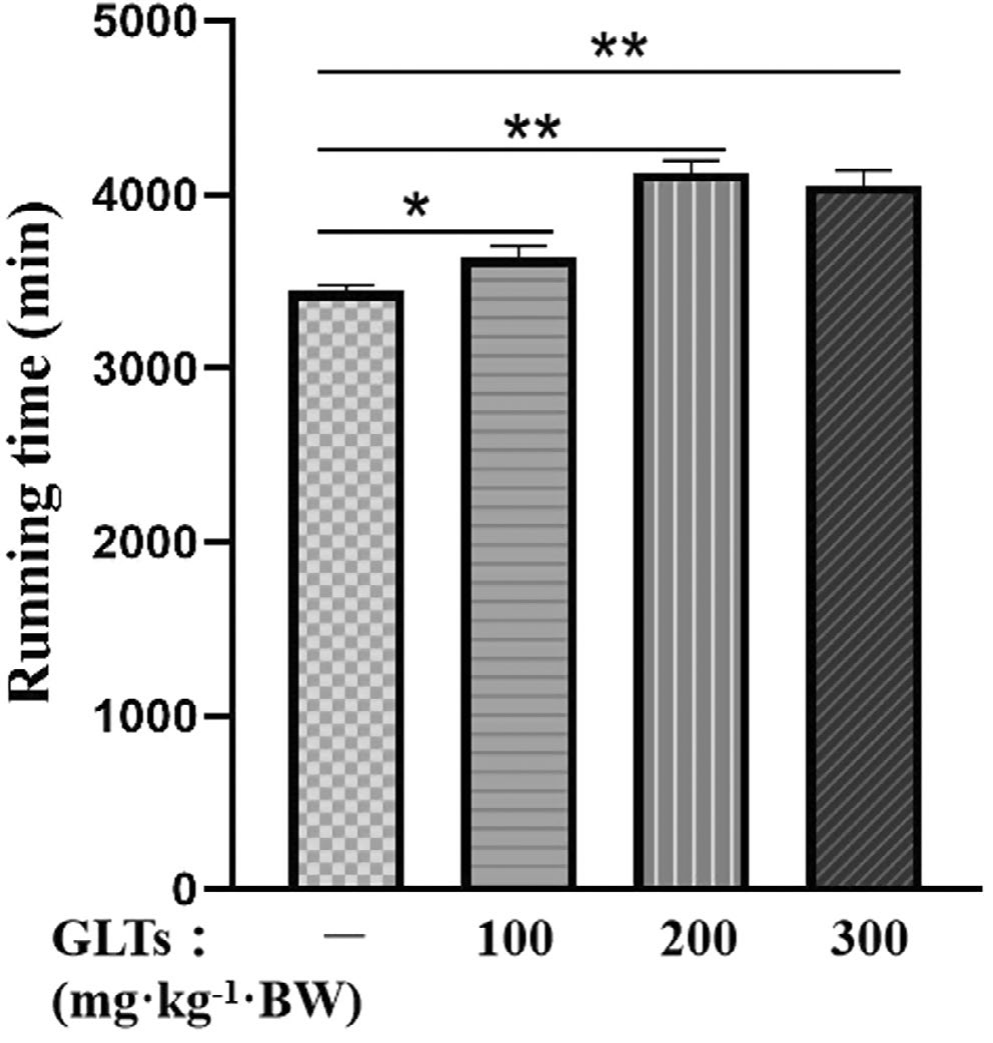
Running exhaustion time of mice in different exhaustive exercise training groups. **p < 0.01 vs. model group. (x ± SD, *n* = 12).

The exhaustive exercise durations of mice in each group are presented in Figure 2. The model group exhibited the shortest duration, whereas the GLTs‐M group demonstrated the longest. Treatment with GLTs at all tested doses significantly prolonged exhaustive exercise time compared to the model group (*p* < 0.01). Among them, the moderate‐dose GLTs group (GLTs‐M) showed the most pronounced improvement. These results highlight the potential of GLTs to enhance exercise capacity and delay fatigue onset, suggesting a dose‐responsive fatigue‐mitigating effect.

### 3.3 Effects of GLTs on Cardiac Function in Exhaustive Exercise Mice

**FIGURE 3:**
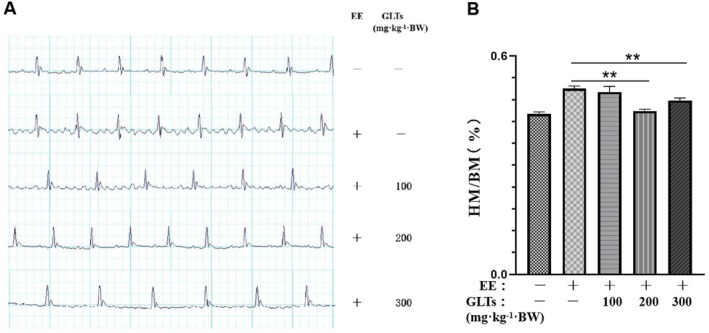
Effect of GLTs on cardiac function of exhaustive exercise training mice. Electrocardiogram examination of mice in each group (A), the cardiac indices of the mice (B). **p < 0.01 compared with the model group. (x ± SD, *n* = 12).

Electrocardiographic (ECG) analysis performed 24 h after the final training session revealed marked cardiac abnormalities (Figure 3A). Mice in the model and GLTs‐L groups exhibited ST‐segment elevation, ST‐T wave abnormalities, and prolonged QT intervals when compared to the control group. These pathological alterations were significantly ameliorated in the GLTs‐M and GLTs‐H groups, demonstrating a dose‐dependent cardioprotective effect of GLTs. Additionally, all exercise groups showed increased PR interval fluctuations, indicative of elevated heart rate and enhanced myocardial contractility. As illustrated in Figure 3B, cardiac indices were significantly increased in all exercise‐exposed groups following 6 weeks of high‐intensity training, reflecting physiological adaptations to exhaustive exercise. GLTs administration mitigated these changes, with the GLTs‐M and GLTs‐H groups showing significantly lower cardiac indices than the model group (*p* < 0.05). No statistically significant changes were observed in the GLTs‐L group (*p* > 0.05), further supporting the dose‐dependent efficacy of GLTs in preserving cardiac morphology under exhaustive exercise conditions.

### 3.4 Effects of GLTs on Myocardial Tissue Morphology in Exhaustive Exercise Mice

**FIGURE 4:**
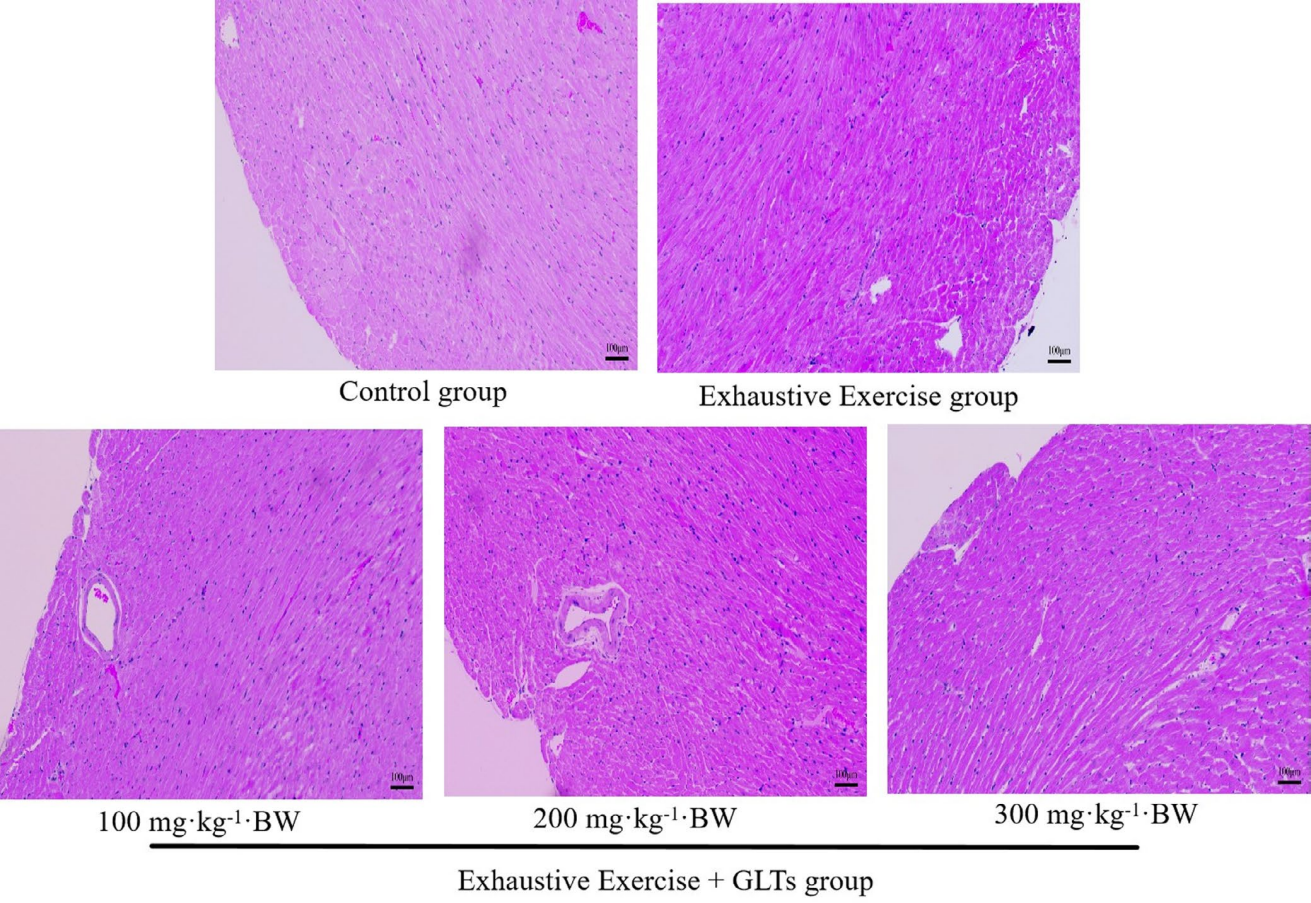
Effect of GLTs on HE staining of mice myocardial tissue. The mice were divided into the control group, the Exhaustive Exercise group, the Exhaustive Exercise + 100 mg/kg GLTs group, the Exhaustive Exercise + 200 mg/kg GLTs group, and the Exhaustive Exercise + 300 mg/kg GLTs group.

Histopathological analysis using H&E staining (Figure 4) revealed notable myocardial injury in the model group, including myofiber disorganization, swelling, necrosis, vacuolization, and interstitial inflammatory infiltration. In contrast, the control group displayed well‐preserved myocardial structure with neatly arranged cardiomyocytes and minimal pathological changes. GLTs administration alleviated these abnormalities in a dose‐dependent manner. Particularly, the GLTs‐M and GLTs‐H groups showed marked improvements in myocardial architecture, including reduced cellular disorganization and inflammatory infiltration. However, mild vacuolization and degeneration persisted compared to the control.

In addition to qualitative observations, semiquantitative scoring (Table 1) was used to assess myocardial damage based on three parameters: myofiber disorganization, necrosis/vacuolization, and inflammatory infiltration. The model group exhibited significantly elevated injury scores (total score: 9.3 ± 0.8) compared to the control (1.4 ± 0.7, *p* < 0.01). GLTs treatment significantly reduced these scores in a dose‐dependent fashion: 6.0 ± 0.8 (GLTs‐L), 4.2 ± 0.5 (GLTs‐M), and 4.1 ± 0.4 (GLTs‐H), with moderate and high doses showing the most pronounced protective effects (*p* < 0.01 vs. model). These findings confirm the efficacy of GLTs in attenuating myocardial injury caused by exhaustive exercise.

**TABLE 1:** Semiquantitative histological scores of myocardial injury in each group.

Group	Myofiber disorganization	Necrosis/vacuolization	Inflammatory infiltration	Total score
Control	0.3 ± 0.5	0.6 ± 0.4	0.5 ± 0.3	1.4 ± 0.7
Model	2.8 ± 0.4	3.4 ± 0.3	3.1 ± 0.6	9.3 ± 0.8
GLTs‐L	2.2 ± 0.6	2.1 ± 0.5	1.7 ± 0.3	6.0 ± 0.8
GLTs‐M	1.4 ± 0.3	1.6 ± 0.2	1.2 ± 0.3	4.2 ± 0.5
GLTs‐H	1.3 ± 0.2	1.8 ± 0.3	1.0 ± 0.2	4.1 ± 0.4

### 3.5 Effect of GLTs on Serum Oxidation Level of Exhaustion Exercise Mice

**FIGURE 5:**
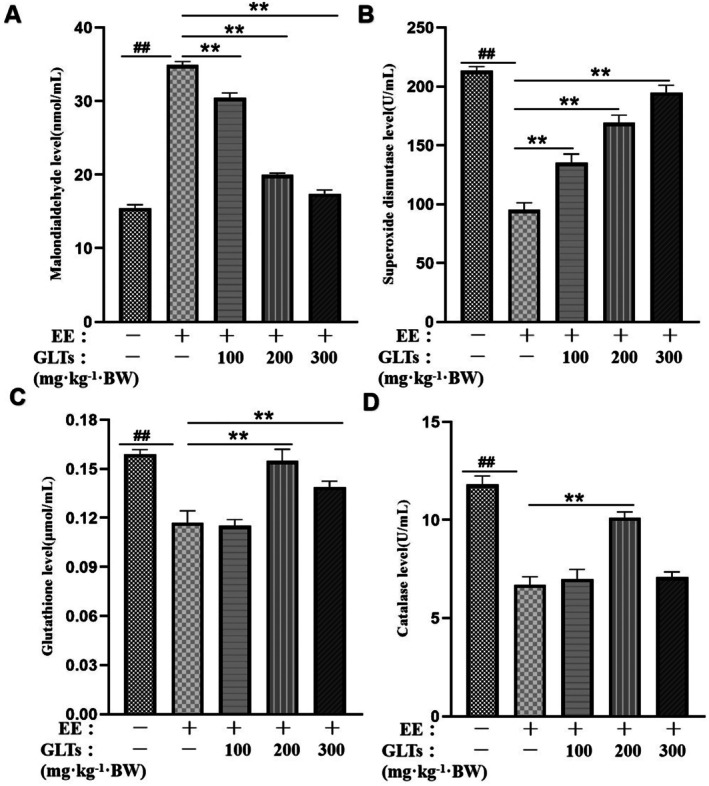
Effect of GLTs on oxidative stress in exhaustive exercise‐induced fatigue in mice. (A) Malondialdehyde (MDA) level. (B) Superoxide dismutase (SOD) level. (C) Glutathione (GSH) level. (D) Catalase (CAT) level. ##*p* < 0.01 compared with the control group. *p < 0.05 compared with the model group. **p < 0.01 compared with the model group. (x ± SD, *n* = 12).

Excessive oxidative stress is a key contributor to fatigue. Thus, we explored the effects of GLTs on oxidative stress induced by strenuous exercise in mice. As shown in Figure 5, serum MDA levels were significantly elevated in the model group compared to the control group, indicating increased oxidative stress. However, GLTs intervention effectively reduced MDA levels in a dose‐dependent manner. Exhaustive exercise also resulted in a significant decline in serum SOD, GSH, and CAT levels. These antioxidant markers were significantly restored in fatigued mice following GLTs administration, demonstrating its protective effects against oxidative stress induced by exhaustive exercise.

### 3.6 Effects of GLTs on Myocardial Apoptosis in Exhaustion Exercise Mice

**FIGURE 6:**
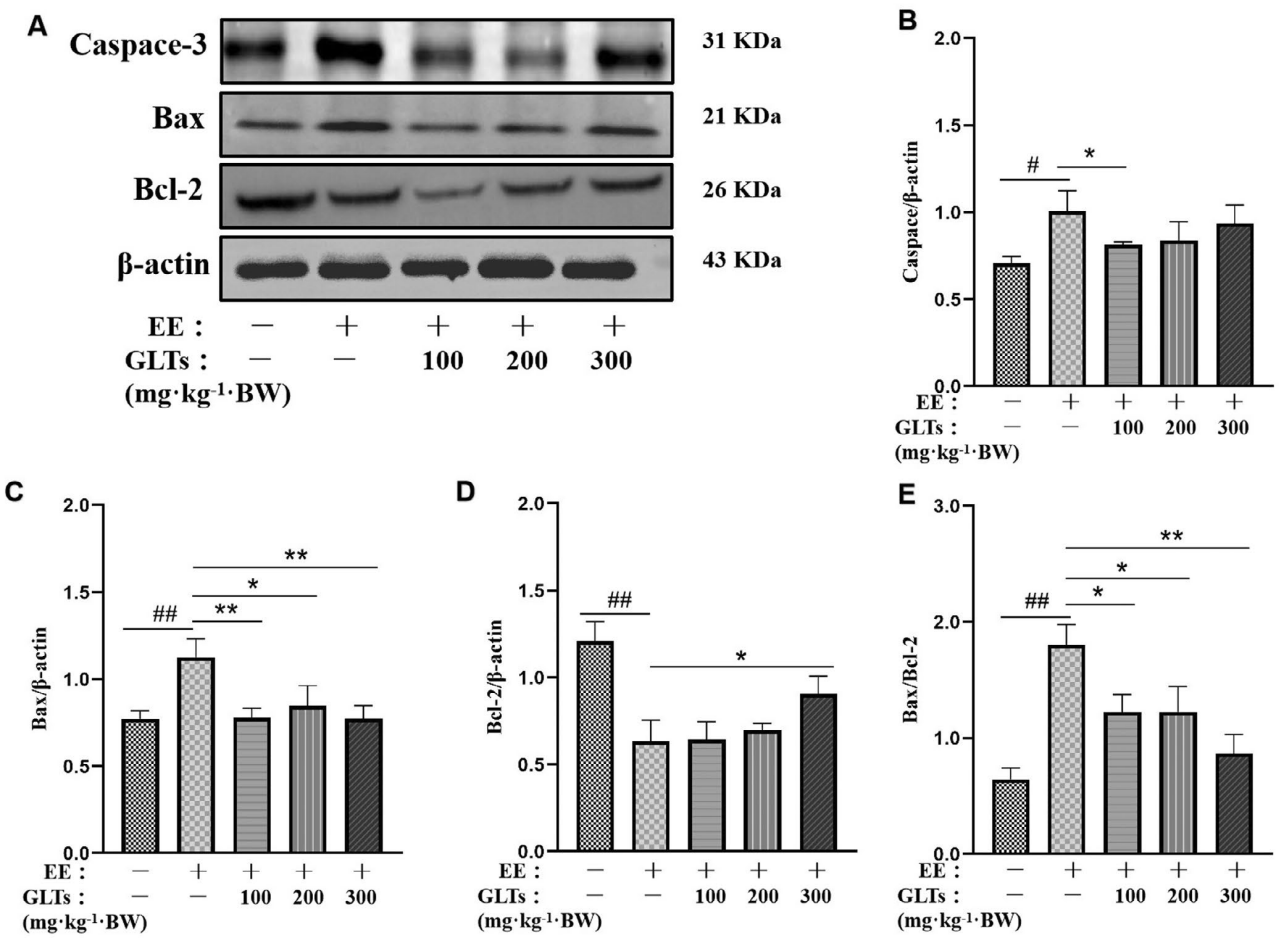
Effect of GLTs on myocardial apoptosis in exhaustive exercise training mice. (A) Representative western blots of Caspase‐3, Bax, Bcl‐2. (B) Quantitation of Caspase‐3. (C) Quantitation of Bax. (D) Quantitation of Bcl‐2. (D) Quantitation of Bax/Bcl‐2. #*p* < 0.05 compared with the control group. ##*p* < 0.01 compared with the control group. *p < 0.05 compared with the model group. **p < 0.01 compared with the model group. (x ± SD, *n* = 12).

Western blot analysis (Figure 6) revealed significant apoptotic changes in mice subjected to exhaustive exercise. Caspase‐3 and Bax levels were markedly elevated, while Bcl‐2 expression was reduced in the model group compared to controls (*p* < 0.05). GLTs intervention significantly lowered Bax levels in all groups (*p* < 0.05), and only the GLTs‐H group showed a notable increase in Bcl‐2 (*p* < 0.05). The Bax/Bcl‐2 ratio, significantly higher in the model group (*p* < 0.01), was reduced across GLTs‐treated groups, with the greatest decrease in the GLTs‐H group (*p* < 0.01), nearing control levels. These results indicate a dose‐dependent antiapoptotic effect of GLTs, with higher doses showing enhanced efficacy.

### 3.7 Effects of GLTs on Cardiac Oxidative Stress Parameters in Exhaustion Exercise Mice

**FIGURE 7:**
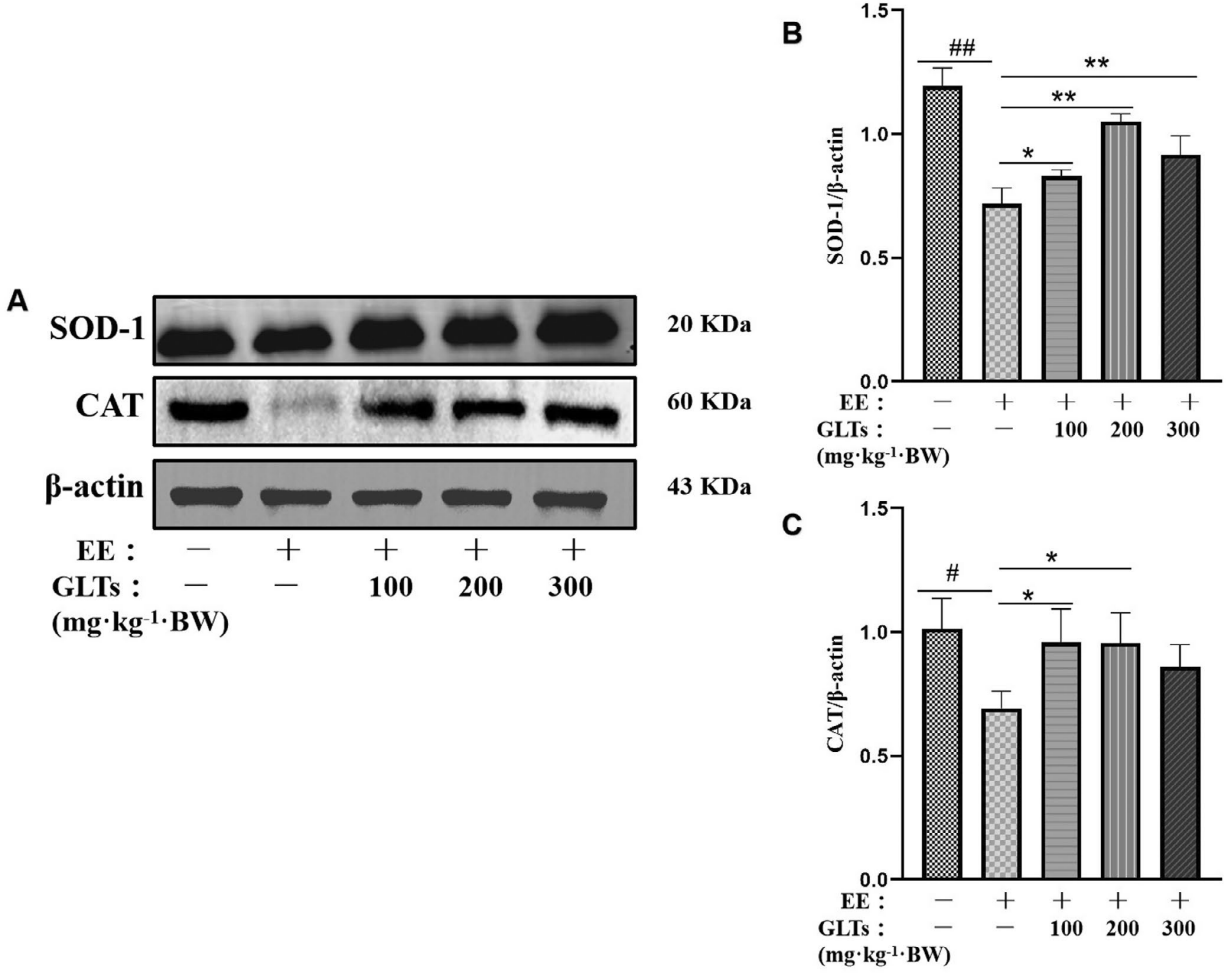
Effect of GLTs on the expression of antioxidant proteins in mice myocardium. (A) Representative western blots of SOD‐1, CAT. (B) Quantitation of SOD‐1. (C) Quantitation of CAT. #*p* < 0.05 compared with the control group. ##*p* < 0.01 compared with the control group. *p < 0.05 compared with the model group. **p < 0.01 compared with the model group. (x ± SD, *n* = 12).

The relationship between oxidative stress markers and the Nrf2 pathway was assessed, with results presented in Figure 7. In the model group, protein expression levels of SOD‐1 and CAT were significantly lower than in the control group (*p* < 0.05), indicating oxidative stress. GLTs intervention effectively increased SOD‐1 expression across all treatment groups (*p* < 0.01). Additionally, GLTs‐L and GLTs‐M groups significantly upregulated CAT expression (*p* < 0.05), while no significant change was observed in the GLTs‐H group compared to the model group (*p* > 0.05). These findings suggest a differential impact of GLTs doses on CAT expression, with low and moderate doses showing greater effectiveness.

### 3.8 Effects of GLTs on Keap1/Nrf2/HO‐1 Signaling Pathway Proteins in Exhaustion Exercise Mice

**FIGURE 8:**
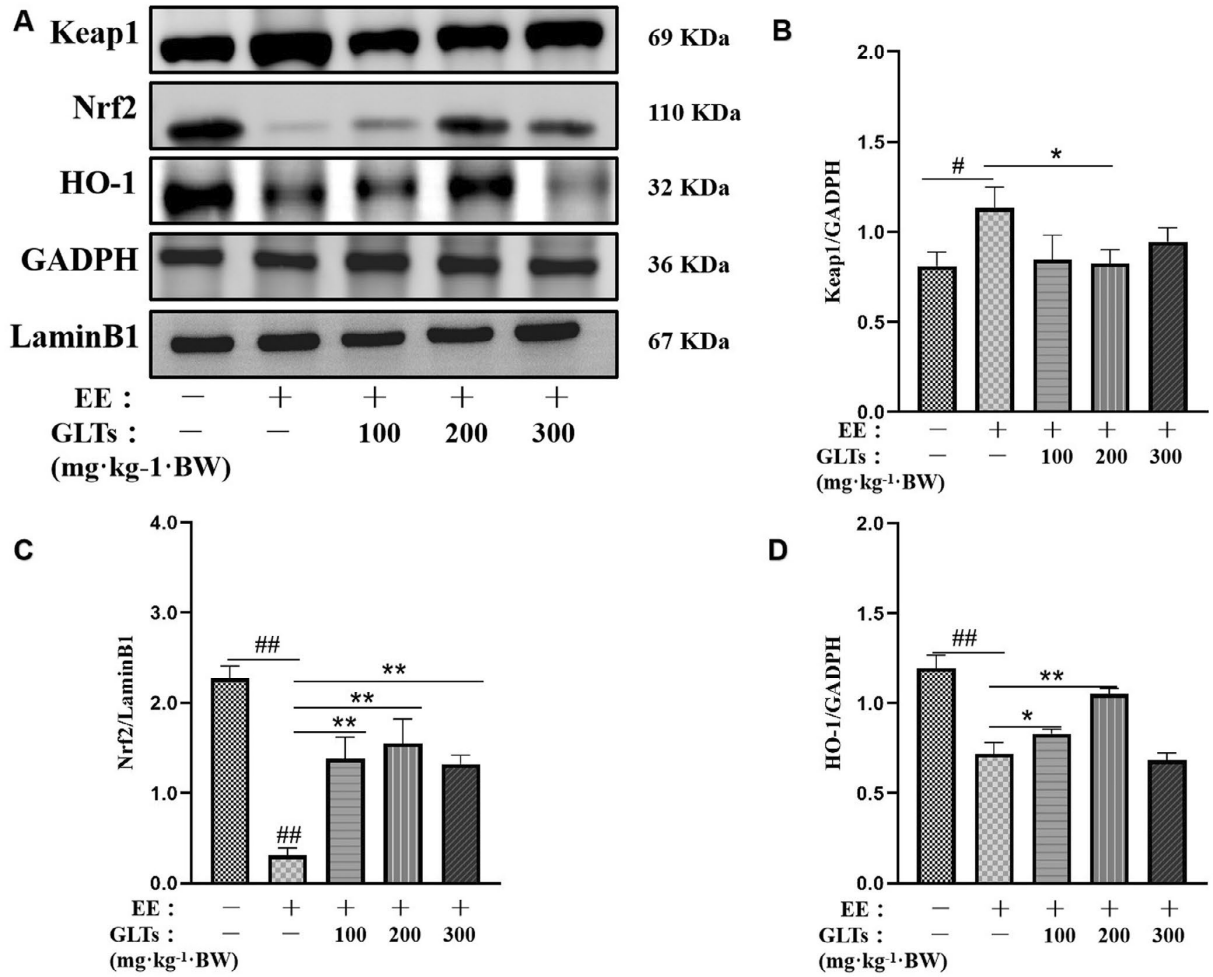
Effects of GLTs on Keap1/Nrf2/HO‐1 signaling pathway protein expression in mice myocardium. (A) Representative western blots of Keap1, Nrf2, HO‐1. (B) Quantitation of cytoplasmic nuclear factor erythroid 2‐related factor 2 (Nrf2). (C) Quantitation of Kelch‐like ECH‐associated protein 1 (Keap1). (D) Quantitation of heme oxygenase‐1 (HO‐1). #*p* < 0.05 compared with the control group. ##*p* < 0.01 compared with the control group. *p < 0.05 compared with the model group. **p < 0.01 compared with the model group. (x ± SD, *n* = 12).

The effects of GLTs on the Keap1/Nrf2/HO‐1 signaling pathway in the myocardium of exhaustive exercise mice are shown in Figure 8A. Keap1 expression was significantly elevated in the model group compared to the control group (*p* < 0.05). All GLTs interventions reduced Keap1 expression, with the GLTs‐M group showing the most significant inhibition of Keap1 expression induced by exhaustive exercise (*p* < 0.05). Additionally, the protein levels of Nrf2 and HO‐1 were significantly lower in the model group than in the control group (*p* < 0.01). After GLTs intervention, both Nrf2 and HO‐1 expression were significantly increased in the GLTs‐treated groups compared to the model group (*p* < 0.05). These findings suggest that GLTs modulate the Keap1/Nrf2/HO‐1 pathway, with the moderate dose (GLTs‐M) showing the most pronounced effect.

## 4 Discussion

Most previous studies on the cardioprotective effects of GLTs have primarily focused on pathological models, including chemically induced myocardial infarction, ischemia–reperfusion injury, or doxorubicin‐induced cardiotoxicity. In these contexts, GLTs were shown to suppress inflammatory responses, attenuate oxidative damage, and inhibit apoptosis via modulation of the NF‐κB, PI3K/Akt, and Nrf2/HO‐1 signaling pathways (Kuok et al. 2013; Samantha et al. 2025; Yarmohammadi et al. 2021). However, such models primarily simulate acute pathological cardiac injury with sustained tissue necrosis and inflammation. In contrast, our study uniquely employs a model of exhaustive exercise myocardial injury, a form of physiological cardiac stress that lacks gross necrosis but is characterized by transient oxidative overload, metabolic imbalance, and functional fatigue. This physiological nature makes it a clinically relevant yet underexplored model, especially for populations undergoing chronic athletic or occupational exertion.

By demonstrating that GLTs supplementation alleviates myocardial structural damage, reduces oxidative stress, and activates the Keap1/Nrf2/HO‐1 pathway in this context, our findings highlight a novel application of GLTs in the field of exercise‐related cardiovascular protection.

Within the spectrum of exhaustive exercise, several biomarkers undergo significant changes, with CK, LDH, and BUN being among the most prominent. These markers reflect the physiological alterations occurring during fatigue (Fukuda et al. 2016). CK, a key enzyme, is known to rise in response to muscle fatigue. Elevated serum CK levels are often used as a clinical marker of muscle damage, with a decrease in CK levels typically indicating recovery from exhaustive exercise injury (Brancaccio et al. 2007). LDH plays a role in converting lactic acid to pyruvate, thus helping to reduce lactic acid buildup in muscle. LDH activity is strongly and positively correlated with the development of fatigue (Farhana and Lappin 2023). Intense physical exertion causes tissue damage, leading to the release of LDH into the bloodstream, which results in elevated lactate dehydrogenase levels (Zhou et al. 2019). Meanwhile, impaired exercise myocardial function elevates LDH. Furthermore, BUN stands as the primary end product stemming from protein metabolism within the human body. Notably, the levels of urea nitrogen in the body exhibit a positive correlation with exercise tolerance, suggesting a potential association between urea nitrogen dynamics and the body's capacity for physical exertion (Shi et al. 2007). An increase in BUN activity generally symbolizes protein catabolism, which will affect muscle contractility and ultimately lead to the onset of fatigue (Bongiovanni et al. 2020). Notably, exhaustive exercise induces significant elevations in CK, LDH, and BUN levels. Our study found that GLTs administration significantly reduced these biomarkers, indicating that GLTs not only expedite the elimination of harmful metabolites but also help restore energy reserves, ultimately alleviating fatigue.

The exhaustion time index is an important measure for assessing the impact of pharmaceutical agents on fatigue and recovery (Yan et al. 2022). In our study, we evaluated the effect of GLTs on fatigue markers. The results showed a significant improvement in fatigue dynamics, with GLTs administration enhancing endurance and delaying fatigue onset in a dose‐dependent manner. These findings highlight the antifatigue effect of GLTs and their potential to improve exercise performance in mice.

Oxidative stress is a key factor in myocardial damage during exhaustive exercise. Intense physical exertion elevates ROS, leading to oxidative damage in cellular structures, including the myocardium. This oxidative stress disrupts myocardial function, contributing to fatigue and muscle injury. Our study suggests that GLTs may protect against myocardial injury by mitigating oxidative stress, as indicated by changes in apoptotic markers. Specifically, oxidative stress induced by fatigue is a known driver of apoptosis, an irreversible physiological process (Sharma et al. 2023). We found that GLTs significantly upregulated the antiapoptotic protein Bcl‐2, downregulated the proapoptotic protein Bax, and reduced caspase‐3 levels. These results provide strong evidence for the protective role of GLTs in preventing apoptosis and myocardial injury induced by intense exercise.

Research has confirmed a connection between exhaustive exercise and the oxidative stress induced by intense physical exertion (Powers et al. 2020). MDA, a major byproduct of free radical‐induced damage, serves as a key marker for assessing cellular oxidative stress, with its presence directly indicating damage to cell membranes (Spirlandeli et al. 2014). One of the primary roles of GSH is scavenging free radicals and other harmful substances, thus protecting cells and tissues from oxidative stress (Papiez et al. 2009). The accumulation of ROS disrupts oxidative balance and antioxidant enzyme activity, contributing to the onset of exhaustive exercise fatigue. Among the body's antioxidant enzymes, SOD plays a critical role in combating oxidative stress, acting as a cornerstone of the enzymatic defense system. It helps to mitigate fatigue and cellular damage by reducing the production of ROS (Ma et al. 2022; Powers et al. 1993). Additionally, CAT is a key enzyme involved in the breakdown of hydrogen peroxide, playing a vital role in oxidative stress protection (Pinho et al. 2006). In our study, GLTs significantly reduced MDA levels while enhancing the activities of SOD, GSH, and CAT. These results suggest that GLTs can enhance antioxidant enzyme activity, thereby decreasing oxidative stress markers, preserving redox equilibrium, and ultimately exhibiting antifatigue effects.

The Keap1/Nrf2/HO‐1 pathway is a crucial antioxidant defense mechanism in animals, with studies indicating its involvement in oxidative damage resulting from excessive exercise (Vargas‐Mendoza et al. 2019; Zhong et al. 2022). In a state of redox homeostasis, Nrf2 is bound to Keap1 in the cytoplasm, keeping it inactive. However, upon oxidative stress or exposure to harmful substances, Nrf2 dissociates from Keap1, translocates into the nucleus, and binds to the ARE, activating the transcription of protective genes such as CAT, SOD, and HO‐1 (Liu et al. 2021; Wu, Liao, et al. 2022). These genes help reduce oxidative stress and protect cells from damage (Meng et al. 2019). The activation of the Nrf2 signaling pathway offers a promising strategy for alleviating fatigue and promoting recovery. In this study, we observed that the expression of the upstream regulator Keap1 in plasma proteins was significantly reduced, while Nrf2 levels were elevated post‐exercise. However, the nuclear expression of Nrf2 and HO‐1 was initially suppressed. Following the intervention with GLTs, we noted a significant increase in the expression of Nrf2 and HO‐1 in the nucleus. This suggests that GLTs can enhance Nrf2's translocation into the nucleus, activating the Nrf2/ARE pathway and boosting the cellular antioxidant response.

Previous studies have demonstrated that Ganoderma lucidum triterpenoids (GLTs) possess protective effects against hepatic injury (Lv et al. 2022; Zhao et al. 2019) and chemically induced cardiotoxicity (Veena and Janardhanan 2022). However, their potential regulatory role in exhaustive exercise myocardial injury has not yet been explored. Exhaustive exercise generates a distinct oxidative stress environment characterized by excessive ROS production, mitochondrial dysfunction, and metabolic imbalance. In this study, we show that GLTs effectively modulate this pathological state by promoting Nrf2 nuclear translocation and enhancing downstream HO‐1 expression. These findings suggest that GLTs exert context‐specific antioxidant effects under exhaustive exercise stress and provide novel mechanistic insights into their cardioprotective potential in physically stressed myocardium.

Furthermore, we observed a clear dose‐dependent effect, with medium and high doses of GLTs significantly enhancing the nuclear expression of Nrf2, HO‐1, and key antioxidant enzymes such as CAT and SOD. This amplified antioxidant response likely underlies the observed myocardial protection. Taken together, our findings indicate that GLTs represent a promising natural intervention for combating oxidative damage and preserving cardiac function under conditions of exhaustive exercise fatigue. These results lay a foundation for further investigation into the application of GLTs in sports medicine and exercise‐related cardiovascular health. However, although this study focused on oxidative stress and apoptotic pathways, future research should incorporate inflammatory markers such as NF‐κB and IL‐6 to comprehensively elucidate the cardioprotective mechanisms of GLTs. In addition, future studies should incorporate additional pathophysiological parameters such as inflammatory cytokines and myocardial fibrosis markers to comprehensively delineate the cardioprotective profile of GLTs.

## 5 Conclusions

This study demonstrates that GLTs exert significant antifatigue and cardioprotective effects in a mouse model of exhaustive exercise. GLT supplementation effectively mitigated oxidative stress, regulated apoptosis‐related proteins, and preserved myocardial structure and function, likely through activation of the Keap1/Nrf2/HO‐1 signaling pathway. These findings underscore the potential of GLTs as a natural antioxidant intervention to alleviate exhaustive exercise myocardial injury and delay fatigue onset. By enhancing redox homeostasis and promoting cellular resilience under physiological stress, GLTs may offer a novel therapeutic strategy for improving endurance and exercise performance. Further preclinical and clinical investigations are warranted to validate their efficacy and safety in managing exercise‐related cardiovascular dysfunction.

## Author Contributions

**Jingrong Li:** Conceptualization (equal), Writing‐original draft (equal), Funding acquisition (equal), Project administration (equal). **Ling Zhang:** formal analysis (equal), validation (equal). **Guangfeng Qin:** conceptualization (equal). **Weiguo Liu:** software (equal). **Xin Xu:** writing – review and editing (equal). **Jialin Zhu:** writing – review and editing (equal). **Shengmei Zhao:** validation (equal). **Taotao Qiu:** conceptualization (lead), funding acquisition (lead), methodology (lead), writing – review and editing (lead).

## Conflicts of Interest

The authors declare no conflicts of interest.

## Data Availability

Data Availability The data that support the findings of this study are available from the corresponding author upon reasonable request.
